# Thermal Stress Interacts With Surgeonfish Feces to Increase Coral Susceptibility to Dysbiosis and Reduce Tissue Regeneration

**DOI:** 10.3389/fmicb.2021.620458

**Published:** 2021-03-25

**Authors:** Leïla Ezzat, Sarah Merolla, Cody S. Clements, Katrina S. Munsterman, Kaitlyn Landfield, Colton Stensrud, Emily R. Schmeltzer, Deron E. Burkepile, Rebecca Vega Thurber

**Affiliations:** ^1^Department of Ecology, Evolution and Marine Biology, University of California, Santa Barbara, Santa Barbara, CA, United States; ^2^Bodega Marine Laboratory, University of California, Davis, Davis, CA, United States; ^3^School of Biological Sciences, Georgia Institute of Technology, Atlanta, GA, United States; ^4^Department of Ecology and Evolutionary Biology, University of Michigan, Ann Arbor, MI, United States; ^5^Department of Microbiology, Oregon State University, Corvallis, OR, United States; ^6^Marine Science Institute, University of California, Santa Barbara, Santa Barbara, CA, USA

**Keywords:** surgeonfish, coral, thermal stress, feces, *Vibrio*, global change, dysbiosis, 16S rRNA

## Abstract

Dysbiosis of coral microbiomes results from various biotic and environmental stressors, including interactions with important reef fishes which may act as vectors of opportunistic microbes via deposition of fecal material. Additionally, elevated sea surface temperatures have direct effects on coral microbiomes by promoting growth and virulence of opportunists and putative pathogens, thereby altering host immunity and health. However, interactions between these biotic and abiotic factors have yet to be evaluated. Here, we used a factorial experiment to investigate the combined effects of fecal pellet deposition by the widely distributed surgeonfish *Ctenochaetus striatus* and elevated sea surface temperatures on microbiomes associated with the reef-building coral *Porites lobata*. Our results showed that regardless of temperature, exposure of *P. lobata* to *C. striatus* feces increased alpha diversity, dispersion, and lead to a shift in microbial community composition – all indicative of microbial dysbiosis. Although elevated temperature did not result in significant changes in alpha and beta diversity, we noted an increasing number of differentially abundant taxa in corals exposed to both feces and thermal stress within the first 48h of the experiment. These included opportunistic microbial lineages and taxa closely related to potential coral pathogens (i.e., *Vibrio vulnificus*, *Photobacterium rosenbergii*). Some of these taxa were absent in controls but present in surgeonfish feces under both temperature regimes, suggesting mechanisms of microbial transmission and/or enrichment from fish feces to corals. Importantly, the impact to coral microbiomes by fish feces under higher temperatures appeared to inhibit wound healing in corals, as percentages of tissue recovery at the site of feces deposition were lower at 30°C compared to 26°C. Lower percentages of tissue recovery were associated with greater relative abundance of several bacterial lineages, with some of them found in surgeonfish feces (i.e., Rhodobacteraceae, Bdellovibrionaceae, Crocinitomicaceae). Our findings suggest that fish feces interact with elevated sea surface temperatures to favor microbial opportunism and enhance dysbiosis susceptibility in *P. lobata*. As the frequency and duration of thermal stress related events increase, the ability of coral microbiomes to recover from biotic stressors such as deposition of fish feces may be greatly affected, ultimately compromising coral health and resilience.

## Introduction

Reef-building corals form associations with a wide array of microorganisms including dinoflagellate algae (“Symbiodinaceae”), bacteria, viruses and archaea, which collectively form the coral holobiont (Rohwer et al., 2002). Interactions between corals and these microbial associates likely play key roles in a number of vital host functions including coral immune response and nutrient cycling (Maynard et al., 2015; McDevitt-Irwin et al., 2017; Robbins et al., 2019; van Oppen and Blackall, 2019). At the same time, coral-associated microbial communities are sensitive to numerous environmental (i.e., temperature, nutrient pollution, and overfishing) and biotic (i.e., corallivory and macroalgal competition) stressors (McDevitt-Irwin et al., 2019). Many of these stressors have been associated with disruptions to coral microbiomes that can lead to microbial dysbiosis (i.e., the loss of beneficial microbes or increase of opportunists) (McDevitt-Irwin et al., 2017; van Oppen and Blackall, 2019), bleaching, and mortality (Hughes et al., 2017) – emphasizing the importance of coral-associated microbial communities to the integrity of reef ecosystems.

Despite the disproportionate role that combinations of stressors are predicted to play as reefs further degrade (Bourne et al., 2016; Hughes et al., 2017; van Oppen and Blackall, 2019), their impacts on coral microbiomes remain poorly understood (Zaneveld et al., 2016; Maher et al., 2019; Rice et al., 2019b). This is especially true for biotic stressors resulting from interactions between corals and reef fishes, such as corallivory (i.e., consumption of living corals) or the deposition of fish fecal material onto corals (Nicolet et al., 2018; Ezzat et al., 2019; Rice et al., 2019a). These interactions are both common and integral to reef ecosystems, but could promote coral dysbiosis and mortality when coupled with anthropogenic stressors (Zaneveld et al., 2016). Identifying and quantifying these microbial interactions will be important for predicting the future dynamics of coral reefs.

Reef fishes play critical and often positive roles in the function and dynamics of coral reef ecosystems (Burkepile and Hay, 2008; Mora, 2015). Herbivores and detritivores remove macroalgae that can harm coral growth, recruitment and survival (Burkepile and Hay, 2008; Rasher et al., 2013), while fish communities more broadly help retain and recycle nutrients within reef ecosystems (Allgeier et al., 2016). Despite these benefits, evidence suggests that a number of species may also harm corals – promoting dysbiosis via transmission or enrichment of opportunistic and/or pathogenic microbial taxa (Chong-Seng et al., 2011; Ezzat et al., 2019). For example, corallivorous butterflyfishes and parrotfishes may spread parasites and bacteria among coral colonies via oral transmission while feeding (Chong-Seng et al., 2011; Ezzat et al., 2020; Noonan and Childress, 2020). Many reef fishes also harbor a diverse gut microbiota that include opportunists and pathogens with the potential to alter coral health (Smriga et al., 2010). A recent study demonstrated that fecal pellets from the surgeonfish *Ctenochaetus striatus* may vector and/or favor the enrichment of opportunistic taxa when they land on coral colonies (Ezzat et al., 2019), possibly leading to tissue mortality when feces sit on corals for prolonged periods.

Negative effects on coral microbiomes due to biotic interactions, such as fecal pellet deposition, may normally be transient (Ezzat et al., 2019; Garren et al., 2009), but such impacts may become more severe as human-induced stressors increase in frequency and intensity (Maynard et al., 2015; McDevitt-Irwin et al., 2019). For instance, rising sea surface temperature is a common abiotic stressor on reefs that can induce shifts in coral bacterial community composition, increase community variability, and promote blooms of opportunistic and pathogenic taxa – all of which are indicative of dysbiosis (Ritchie, 2006; Bourne et al., 2016; McDevitt-Irwin et al., 2017; Maher et al., 2019). Elevated sea surface temperature could therefore interact with fish fecal deposition to enhance the growth and virulence of specific microbes, including potential pathogens (i.e., *Vibrio, Photobacterium*) that could compromise coral immunity, health and resilience.

In light of our previous work (Ezzat et al., 2019), this study investigated how fish feces deposition impacted coral microbiomes and tissue regeneration when corals also experienced thermal stress. Working in Mo’orea, French Polynesia, we focused on the line bristletooth surgeonfish *Ctenochaetus striatus*, a functionally important and widely distributed detritivore known to produce substantial amounts of fecal pellets that frequently land on corals (Krone et al., 2008) and can disrupt coral microbiomes (Ezzat et al., 2019). Coral reefs in Mo’orea have been experiencing periodic bleaching events since the 1990’s, with average seawater temperatures fluctuating from 26°C up to 30°C during periods of thermal stress related to bleaching (Pratchett et al., 2013). Therefore, using a factorial designed experiment, we monitored temporal changes in the diversity, stability and compositionality of bacterial communities on massive *Porites lobata* corals exposed to *C. striatus* feces under ambient and elevated sea surface temperatures. In addition, we tracked the recovery of *P. lobata* bacterial communities and rates of tissue regeneration following removal of fish feces from coral tissues when exposed to either thermal condition. We hypothesized that the diversity and variability of *P. lobata* microbiomes would increase when exposed to either stressor alone and would exceed these individual effects when stressors were combined, thus acting synergistically. Specifically, we hypothesized that the combination of fish feces and temperature would (i) shift bacterial assemblages toward greater abundances of opportunistic bacteria involved in bleaching and coral diseases (e.g., *Vibrio* spp.), and (ii) prolong the persistence of bacterial opportunists following removal of fish fecal pellets from a coral’s surface. Finally, we hypothesized that (iii) following fecal pellet removal, feces-induced coral lesions would exhibit lower percentage of tissue recovery when exposed to elevated temperature.

## Materials and Methods

### Sample Collections

The present study was conducted in Mo’orea, French Polynesia (17° 29′ 26.0″ S, 149° 49′ 35.10″W) in August 2018 and based on the previous work by Ezzat et al. (2019). Briefly, thirty-two *Porites lobata* colonies (∼20 cm diameter) were collected at 3 m depth in the back reef area along the north shore of Mo’orea, immediately stored in coolers, and transported back to the Gump South Pacific Research Station. At the station, coral colonies were immediately placed in eight 150 L mesocosms (*n* = 4 colonies per mesocosm) and exposed to comparable light intensity (800 μmol photons m^–2^ s^–1^) and temperature (26°C ± 1°C) regimes. Mesocosms received a continuous supply of seawater from the nearby reef at a flow rate of ∼ 30L h^–1^. Submersible pumps were placed in each mesocosm to ensure proper water mixing.

A total of 15 individuals of *Ctenochaetus striatus* (∼ 20 cm total length) were collected at ∼ 3 m depth within the same back reef area using hand and barrier nets. Fish were then euthanized and dissected to retrieve feces samples from the lowest part of their intestines (last 3–7 cm before the anus). Feces samples were then homogenized in a sterile Whirl-Pak and directly placed on coral fragments as described below. Fish collections, as well as feces sampling, processing, and placement on corals, were completed on the same day.

### Experimental Design of Feces and Temperature Interactions

**FIGURE 1:**
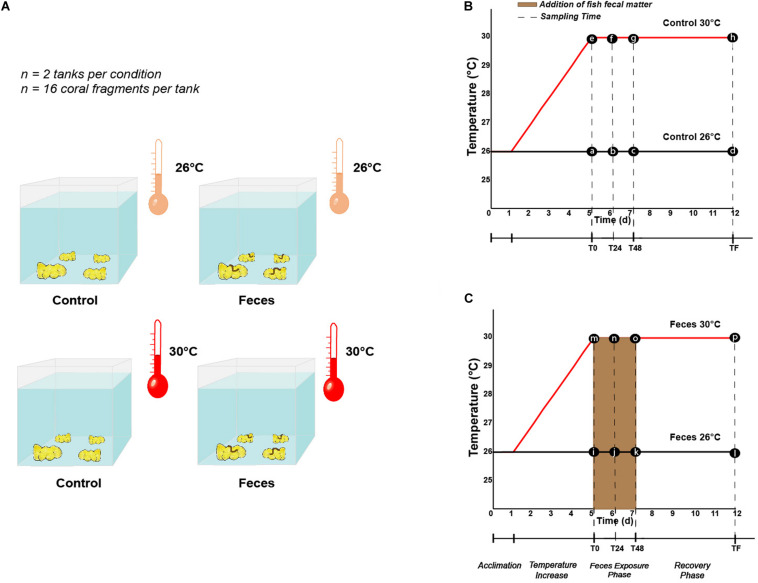
**(A)** Illustration describing the different treatments (Control vs Feces) and temperature regimes (26°C vs 30°C) in which *Porites lobata* were exposed (Illustration by Nathalie Técher). Experimental design illustrating the temperature regimes (26°C and 30°C), the different phases (exposure and recovery) and specific time points at which **(B)** control corals and **(C)** corals exposed to fish feces were sampled (dashed lines). Letters respectively stand for (a) Control T0 26°C, (b) Control T24 26°C, (c) Control T48 26°C, (d) Control TF 26°C, (e) Control T0 30°C, (f) Control T24 30°C, (g) Control T48 30°C, (h) Control TF 30°C, (i) Feces T0 26°C, (j) Feces T24 26°C, (k) Feces T48 26°C, (l) Feces TF 26°C, (m) Feces T0 30°C, (n) Feces T24 30°C, (o) Feces T48 30°C, (p) Feces TF 30°C.

Each *P. lobata* colony was cut into 4 coral fragments using a band saw, to produce a total of 128 fragments that were ∼ 8 cm diameter. Coral fragments were then evenly sorted into 8 mesocosms, so that one fragment of each colony was placed in each treatment (*n* = 2 mesocosms per treatment, *n* = 4 treatments, *n* = 16 coral fragments per mesocosm). Each fragment was then allowed to recover for 24 h prior to any change in temperature regime. Following the recovery period, four mesocosms remained at 26°C ± 1°C while seawater temperatures in the four remaining mesocosms increased from 26°C ± 1°C to 30°C ± 1°C over a 4 day-period (1°C per day). At the end of the fourth day, mesocosms were designated both by treatment (Control vs Feces) and temperature conditions (26°C vs 30°C) (Figure 1A): (1) tanks with coral fragments exposed to ambient seawater temperature (Control 26°C), (2) tanks where a fish fecal pellet was deposited on each coral fragment and exposed to ambient seawater temperature (Feces 26°C), (3) tanks with coral fragments exposed to elevated seawater temperature (Control 30°C), and (4) tanks where a fish fecal pellet was deposited on each coral fragment and exposed to elevated seawater temperature (Feces 30°C). The experiment commenced (T0) upon placement of fecal pellets on all designated coral fragments (∼ 300 mg per fragment).

Four coral fragments were randomly sampled from each mesocosm at three different timepoints during our experiment: (1) immediately after placing fecal pellets on all designated coral fragments (T0), at 24h (T24), and 48h (T48) (Figures 1B,C). Upon collection, all coral fragments were rinsed with a turkey baster using 0.2 μm filtered seawater to remove residual fecal matter from exposed corals. Then, using a sterilized bone cutter, a 1 × 1 cm portion of coral tissue (mucus, tissue and part of the skeleton) was sampled where the treatment was applied (feces deposition) or at a comparable location on corals lacking feces. All samples were then placed in separate (or individual) Whirl-Pak(s). At T48, all remaining coral fragments (*n* = 8 per treatment) were rinsed as mentioned above and placed back in their respective mesocosm to assess recovery of the corals’ microbiomes (hereafter called “recovery phase”) and rate of coral tissue regeneration among corals subjected to fecal pellet deposition (see Ezzat et al., 2019 and the paragraph below). This 48 h timepoint was chosen to reflect the residence time of *C. striatus* fecal pellets on corals previously observed in the field (Ezzat et al., 2019). At the end of the experiment (TF, 1 week), the remaining coral fragments (*n* = 8 per treatment) were collected (Figures 1B,C) and coral tissues sampled as described above. In addition, one 1L sample of seawater was collected from each mesocosm at each timepoint and directly filtered onto a 0.2 μm filter (MilliporeSigma) to compare their microbial communities to the ones of coral samples from the same mesocosm. Finally, three samples of surgeonfish feces were collected following dissection of the same group of fishes and directly disposed in independent Whirl-Pak bags to compare their bacterial communities to those of coral samples and establish potential route of transmission and/or enrichment. Coral tissue, filter and fish feces samples were then transferred into bead tubes (MoBio/Qiagen Power Soil) and stored at -80°C until processing.

### Percentage of Coral Tissue Recovery

We previously observed that deposition of surgeonfish fecal pellets onto *P. lobata* coral fragments induced apparent bleaching and subsequent tissue loss (Ezzat et al., 2019). Thus, in the present study, we monitored the percentage of tissue recovery between T48 and TF (1 week) on coral fragments that were initially exposed to fecal pellets (Feces 26°C and Feces 30°C). Photographs of coral fragments previously exposed to fecal pellets were taken at both T48 and TF. In each case, we measured the surface area (SA) of coral bleaching using ImageJ (Schneider et al., 2012). To quantify tissue recovery after 5 days, we calculated the difference between the initial and final lesion size as a percentage of the initial lesion size: (SA_T__48_ – SA_TF_)/(SA_T__48_)^∗^100.

### DNA Extraction and 16 rRNA Gene Amplification

DNA extraction was performed on coral tissue, filters and feces samples using DNeasy PowerSoil Kit (Qiagen) following the manufacturer’s instructions. High-throughput sequencing of the 16S rRNA gene was performed to compare the diversity, composition and stability metrics of bacterial communities. AccuStart II PCR ToughMix PCR reagent (Quanta BioSciences, Gaithersburg, Maryland, USA) was used in a two-step method to amplify and tag the V4 hypervariable region of the 16S rRNA gene using the primers 515F (5′GTGYCAGCMGCCGCGGTAA-3′) (Parada et al., 2016) and 806R (5′-GGACTACNVGGGTWTCTAAT-3′) (Apprill et al., 2015) targeting bacterial and archaeal communities. The reaction included 6.25 μl AccuStart II ToughMix (2X), 1.25 μl forward primer (10 μM), 1.25 μl reverse primer (10 μM), 0.5 μl sample DNA, and 3.25 μl PCR-grade water. PCR amplification followed a 3 min denaturation at 94°C; 35 cycles of 45 s at 94°C, 60 s at 50°C, and 90 s at 72°C; ending with 10 min at 72°C. To avoid host DNA contamination, amplified samples were run on a 1.5% agarose gel and each visible 16S band was individually poked up to five times with a sterile 10 μl plastic pipette tip. In this way, we ensured that the majority of barcoded product was the 16S V4 region target for the 2nd-step amplification. Tips were then placed into separate PCR tubes already prepared with second-step barcoding master mix solution (12.5 μl ToughMix (2X), 9.5 μl water, and 1 μl of gel-purified sample DNA), swirled in solution to incorporate, and then removed. Following addition of 1 μl each of custom forward and reverse multiplexing barcodes, the 12-cycle barcoding reaction followed a 5 min denaturation at 95°C, 30 s melting at 95°C, 3 min annealing at 63°C, 30 s extension at 72°C, and 10 min hold at 72°C. Barcoded amplicons were pooled in equivolume ratios and purified with Agencourt^®^ AMPure XP beads. Relative libraries were submitted to the Center for Genome Research and Biocomputing (CGRB at Oregon State University (OSU) for sequencing on the Illumina MiSeq Platform (2 × 300bp paired-end reads, MiSeq v.3).

### Amplicon Sequence Data Processing and Quality Control

A total of 166 amplicon sequence libraries were generated, including those from coral tissue, fish feces, water filters and negative control samples (i.e., no-template-controls from PCR). 16S rRNA gene V4 amplicon sequences were processed using Quantitative Insights into Microbial Ecology 2 (QIIME2^1^; v. 2019.9; Bolyen et al., 2019). Plugin demux was used to visualize interactive quality plots and assess read quality. Paired-end sequencing generated a total of 2,828,626 reads across 166 samples. Raw sequences were first trimmed of primers with plugin cutadapt (Martin, 2011). Using DADA2 (Callahan et al., 2016), sequences were truncated for poor-quality bases (quality score < 35), chimeras were filtered, and paired-end reads were merged. This process resulted in a final dataset of 1,298,170 reads across 162 samples, after four samples were discarded in the workflow including one coral sample exposed to feces and the negative controls. Taxonomy was assigned against the SILVA reference database (v.123) (Quast et al., 2012), using classify-sklearn algorithm in QIIME2 (Bokulich et al., 2018). Mitochondria, chloroplasts and host DNA related reads were filtered out using the taxa plugin, further resulting in a total of 1,196,579 reads across 162 samples. A phylogenetic tree was processed with the plugins alignment and phylogeny for further downstream analyses. The biom table harboring the taxonomic counts, the phylogenetic tree and metadata were imported in R (v.3.6.1) for further statistical analyses. Rarefaction curves (Species richness and Shannon-Wiener indices) were generated (Supplementary Figure 1) and rarefaction level was set at 1317 reads per sample, after 20 samples were discarded due to low sampling depth. This included 8 coral samples exposed to control treatment, 11 coral samples exposed to fish fecal treatment and one water sample.

### Amplicon Sequence Data Analytics

The following statistical analyses were performed in R (v.3.6.1). Two alpha diversity metrics were computed, including the observed richness and the Shannon-Wiener indices. Using linear mixed effect (LME) models with the package lme4 (v.1.1-21), the effects of the “treatment” (Control vs Feces), “temperature” (26°C vs 30°C), sampling “time” for the exposure (T0, T24, T48) and recovery phases (T48, TF) as well as their interactions on alpha diversity metrics were assessed. Models were fitted using the restricted maximum likelihood, including “treatment,” “temperature,” and “time” as well as their factorial interactions as fixed factors, while individual “tank” and “colony” were treated as random factors, to take into account the fact that multiple fragments of each treatment were present in each tank. When significant, pairwise comparisons among group levels were processed using the least square means (LSM) present within the package lmerTest (v. 3.1-1). Data residuals were tested for normality and homoscedasticity using Shapiro-Wilk and Levene Tests.

To illustrate the relative abundance of the 25 most abundant amplicon sequence variants (ASVs) as a function of the “treatment,” “temperature,” and “time” and across the whole dataset, we computed a heatmap and agglomerated the taxa at the family level using the function tax_glom (including the command NArm = F) within the phyloseq package (v.1.30). To assess the effects of “treatment”, “time” and “temperature” on the relative abundance of the most abundant family in the coral dataset (i.e., Endozoicomonadaceae), we used a LME as previously described and agglomerated taxa at the family level.

To display shifts in bacterial community composition as a function of the “treatment,” “temperature,” and “time” within the exposure and recovery phases, two distinct principal coordinate analyses (PCoA) were computed on the Bray-Curtis dissimilarity matrices (999 permutations) using the function plot_ordination within the phyloseq package. Analyses of variance (PERMANOVA) based on the Bray-Curtis dissimilarity matrices were used to test for changes in community composition (beta diversity) as a function of (i) the “treatment,” “temperature,” “time,” “tank,” and “colony”, (ii) “treatment,” “temperature,” and “sampletype” (coral vs water samples), “tank” within the exposure and recovery phases, with the function adonis in the package vegan. Pairwise differences were tested using the function pairwise.adonis in the package vegan (v.2.5-6). *P*-values were adjusted according to the Bonferroni method, which accounts for multiple comparisons.

Bacterial composition variability was tested using an analysis of multivariate homogeneity of group dispersions as follows: (i) between treatments (Control vs Feces) within a specific temperature and sampling time (T0, T24, T48 and T48, TF), (ii) between temperatures within a specific treatment and a specific timepoint. For this, we used the function betadisper in the package vegan on the Bray-Curtis dissimilarity matrices. When significant, pairwise tests were performed between groups using Tukey HSD.

The package DESeq2 (v.1.26) was used on the unrarefied ASV table to assess the differential abundance of ASVs as follows: (i) across treatments within a specific time period and temperature, (ii) within a specific treatment, across temperature and within a specific time period. DESeq2 includes a model based on the negative binomial distribution and a Wald’s *post hoc* test for significance testing. *P*-values were adjusted according to the Benjamin and Hochberg method (Benjamini and Hochberg, 1995), accounting for multiple comparisons.

We assessed the presence of indicator ASVs for corals in the different treatments, within each time period and temperature at the species level within the indicspecies package (v.1.7.8). The resulting list of indicator ASVs was then screened to highlight bacterial groups previously described as potential coral pathogens.

Furthermore, the effect of temperature on the percentage of coral tissue recovery was tested using an ANOVA with “temperature” as factor at TF (1 week). Finally, in order to detect associations between specific individual ASVs (from the coral dataset and at the genus level) and the percentage of coral tissue recovery, we deployed a series of linear models in R, linking the percentage of tissue recovery to the relative abundance of each ASV in turn, and allowing for varying intercepts between temperature regimes. Given the relatively low sample size (*N* = 13), the probability of type II error is high even for moderate to high effect sizes (Cohen, 1998). As a consequence, we chose to focus on model fit as measured by the coefficient of determination (*R*^2^). We ranked ASVs according to the proportion of variance in tissue recovery explained by their relative abundance – i.e., by decreasing *R*^2^. Since there is no objective method for distinguishing “high” from “low” correlation, we looked for discontinuities in the distribution of *R*^2^, expressed as departures from zero in the distribution of empirical second derivatives. We examined the biological relevance of ASVs that correlate more strongly with tissue recovery than expected in a smooth distribution, in other words ASVs whose *R*^2^ values are higher than the discontinuity threshold in the ranked *R*^2^ distribution. Normality and homoscedasticity of the data residuals were tested using Shapiro-Wilk and Bartlett tests. All data presented in the result section are described as mean ± standard error.

## Results

### Feces Exposure and Time Altered the Relative Abundance of the Dominant Symbiont Endozoicomonadaceae

**FIGURE 2:**
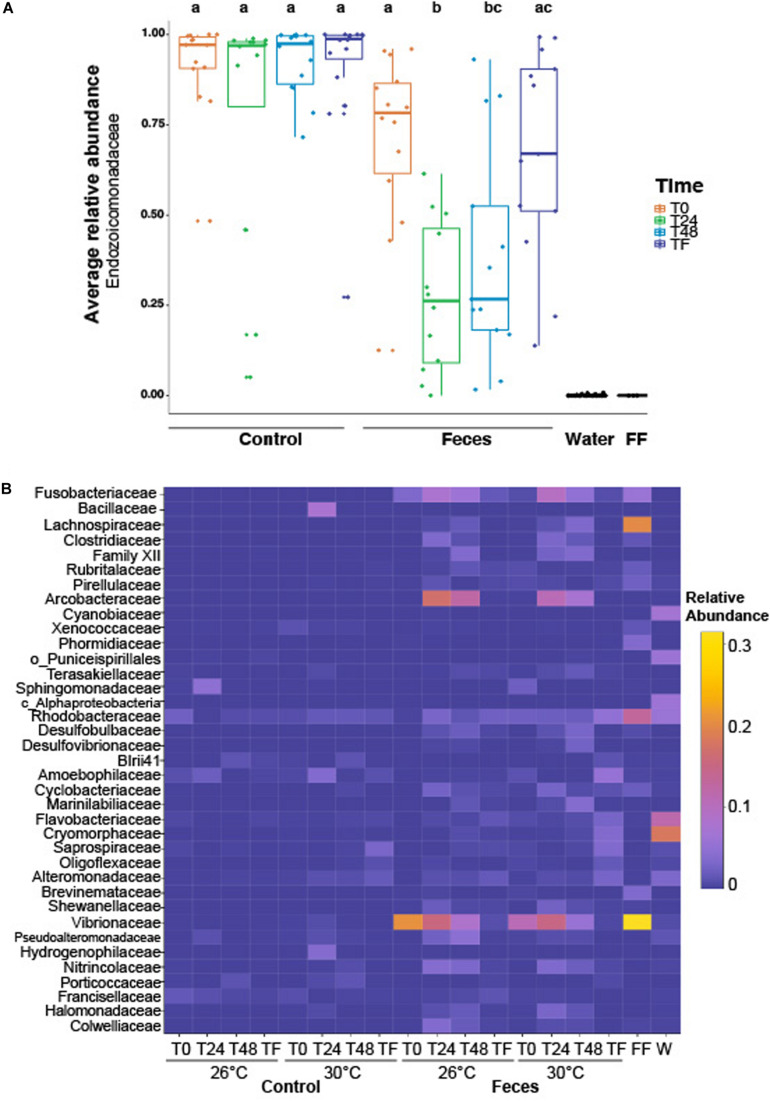
**(A)** Boxplot illustrating the relative abundance of the most abundant family across the whole dataset (i.e., Endozoicomonadaceae), according to experimental treatments (feces vs control), time periods (T0, T24h, T48h, and TF). Lowercase letters above the plot represent the statistical significances based on pairwise comparisons (*p* < 0.05) computed on the coral dataset. The average relative abundance is based on *n* = 12–16 samples per condition. **(B)** Heatmap representing the relative abundance of the remaining abundant families (not including Endozoicomonadaceae) labeled as family, order or class across the whole dataset, according to experimental treatments, time periods and temperatures. “FF” and “W” stand for fish feces and water samples respectively. The average relative abundance is based on *n* = 6–8 samples per condition.

Coral microbiomes were populated by 254 families across all phyla. Only a few families harbored a relative abundance > 1%, including: Endozoicomonadaceae (71% ± 0.03), Vibrionaceae (4.7% ± 0.01), Arcobacteraceae (2.9% ± 0.006), Fusobacteriaceae (2.01% ± 0.003) and Rhodobacteraceae (1.63% ± 0.001) (Supplementary Table 1). Treatment and time significantly affected the average relative abundance of the most abundant family, regardless of temperature (Endozoicomonadaceae; Figure 2A and Supplementary Table 2; *p*_treatment__*time_ = 0.006). We observed a significant decrease in the relative abundance of Endozoicomonadaceae in microbiomes of corals exposed to fish feces at T0 compared to T24 (Figure 2A and Supplementary Table 2; *p* = 0. 001) and a significant increase between T24 and TF (Figure 2A and Supplementary Table 2; *p* = 0.004). Microbiomes of corals exposed to fish feces showed a lower relative abundance of Endozoicomonadaceae at T24 and T48 compared to control corals at all time points (Supplementary Table 2; *p* < 0.04 for all tests).

### Bacterial Assemblages in *P. lobata* Differed From Tank Water and Fish Feces

Bacterial assemblages in tank water were populated by Proteobacteria, Bacteroidetes and Cyanobacteria. Family-level assignment showed the predominance of, among others, Cryomorphaceae (18.2% ± 0.03), Flavobacteriaceae (11.8% ± 0.01), Cyanobiaceae (6.9% ± 0.006), Rhodobacteraceae (5.8% ± 0.005) across a total of 331 families (Figure 2B and Supplementary Table 3).

The bacterial assemblages in surgeonfish feces samples were dominated by members from the phylum Proteobacteria, Firmicutes and Fusobacteria. Across all phyla, 60 families were detected and only a few of them were present at a relative abundance > 2%, including Vibrionaceae (31.5% ± 0.15), Lachnospiraceae (20.1% ± 0.04), Rhodobacteraceae (13.2% ± 0.08), Fusobacteriaceae (5.8% ± 0.03), Phormidiaceae (3.6% ± 0.04), Brevinemataceae (3.4% ± 0.02), Pirellulaceae (2.3% ± 0.02) (Supplementary Table 4).

We found that the composition of bacterial assemblages in *Porites lobata* significantly differed from assemblages in tank water and in *C. striatus* feces (Figure 2B and Supplementary Table 5; F = 33.9; p = 0.003 for all tests). In addition, microbial assemblages associated with corals were different from tank waters throughout the exposure and recovery phases, across treatments and regardless of temperature (Figure 2B and Supplementary Table 6; *F* = 4.2, *p*_sample:treatment_ = 0.02, *p* < 0.01).

### Feces Exposure and Time Increased Bacterial Richness and Diversity

There was a significant interaction between treatment and time on both observed richness (Supplementary Figure 2A and Supplementary Table 7; *F*-value = 7.9; *p* < 0.001) and Shannon-Wiener indices (Supplementary Figure 3A and Supplementary Table 8; *F*-value = 9.9; *p* < 0.001) throughout the exposure phase (between T0 and T48). This pattern was present regardless of temperature (Supplementary Tables 7, 8). Corals exposed to feces had consistently greater values of ASV richness and Shannon-Wiener indices compared to control corals (Supplementary Table 9; *p*_richness_ < 0.03 for all tests; Supplementary Table 10; *p*_diversity_ < 0.001 for all tests). In addition, while control corals showed similar values of richness (Supplementary Table 9; *p* > 0.47 for all tests) and diversity (Supplementary Table 10; *p* > 0.59 for all tests) throughout the exposure phase, corals exposed to feces exhibited greater values of richness and diversity at T24 and T48 compared to T0 (Supplementary Table 9; *p*_richness_ < 0.001; Supplementary Table 10; *p*_diversity_ < 0.001 for all tests respectively).

During the recovery phase (T48-TF), there was a significant effect of the treatment only on both observed richness (Supplementary Figure 2B and Supplementary Table 11; *F*-value = 27.8; *p* = 0.003) and Shannon-Wiener indices (Supplementary Figure 3B and Supplementary Table 12; *F*-value = 42.5; *p* < 0.001), with control corals characterized by lower values of richness and diversity compared to corals exposed to feces.

### Exposure to Feces and Time Altered Bacterial Assemblages in *P. lobata*

We observed a significant interaction between treatment and time on bacterial community composition throughout the exposure phase (Supplementary Figure 4 and Supplementary Table 13; PERMANOVA, *F*-value = 2.31; *R*^2^ = 0.05; *p* = 0.015) and recovery phases (Supplementary Figure 5 and Supplementary Table 14; PERMANOVA, *F*-value = 2.99; *R*^2^ = 0.04; *p* = 0.009). At the beginning of the experiment (T0), control corals exhibited similar bacterial assemblages compared to corals exposed to feces (Supplementary Table 16; *p* = 0.11). Bacterial communities in control corals and corals exposed to feces were mainly dominated by sequences from the family Endozoicomonadaceae (88.7% and 71.5% respectively; Supplementary Table 16). However, bacterial assemblages differed between control corals and corals exposed to feces at T24 (Supplementary Table 15; *p* = 0.015) and T48 (Supplementary Table 15; *p* = 0.015). Specifically, coral microbiomes exposed to feces were mainly populated by members from the families Endozoicomonadaceae, Vibrionaceae, Arcobacteraceae, Rhodobacteraceae, Fusobacteraceae, Flammeovirgaceae, Colwelliaceae, and Nitrincolaceae at T24 and T48 respectively (Supplementary Table 16). In contrast control corals were mainly dominated by Endozoicomonadaceae throughout the exposure and recovery phases and few additional microbes were present above the detection level (Supplementary Table 17).

At the end of the recovery phase (TF), bacterial assemblages exposed to feces shifted back to their original composition and were similar to assemblages in control corals (Supplementary Table 18; *p* = 0.11), with microbiomes of corals mainly dominated by taxa from the family Endozoicomonadaceae (Supplementary Table 16). While control corals showed similar composition in their bacterial assemblages throughout the duration of the exposure and recovery phases (Supplementary Tables 15, 19; *p* = 1 for all tests), corals exposed to feces exhibited distinct composition at T0 compared to T24 (Supplementary Table 15; *p* = 0.015) and T48 (*p* = 0.015) respectively, as well as T48 compared to TF (Supplementary Table 18; *p* = 0.04).

### Feces Exposure Interacted With Temperature to Form Novel Coral Microbiomes

#### Comparison of the Treatments Within Each Time Period and Temperature

**FIGURE 3:**
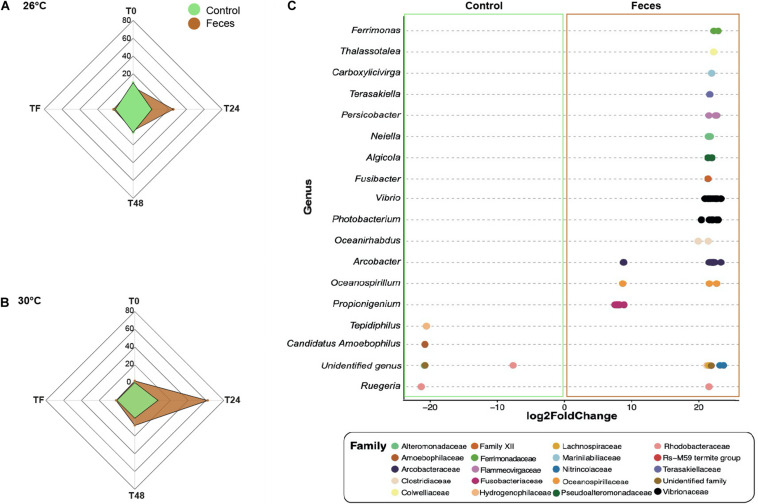
Differential abundance analyses based on DESeq2. Number of differentially abundant ASVs observed in control corals compared to corals exposed to feces within each time point (T0, T24, T48, and TF) at **(A)** 26°C and **(B)** 30°C. **(C)** The ASVs labeled as family and genus that differed significantly between corals exposed to feces and control corals at T24 and under 30°C. Positive values are associated with differentially abundant taxa observed in corals exposed to feces.

For clarity, all results presented within this section applied to corals exposed to feces and included ASVs with an adjusted *p* < 0.05. Additional results regarding control corals can be found in the Supplementary Material (Supplementary Text 1). At T0 and under normal temperature (26°C), corals exposed to feces showed a greater abundance of five taxa from the families Vibrionaceae (e.g., ASVs_4496, 4579) (Figure 3A and Supplementary Table 19; log2 fold change: from 7.6 to 21.9) compared to control corals. At T24, the number of differentially abundant taxa in corals exposed to feces reached 25 ASVs compared to control corals (Figure 3A; log2 fold change: from 6.7 to 22.8). This included sequences from the genera *Propionigenium* (ASVs_200, 207-212), *Arcobacter* (e.g., ASVs_1357, 1371) and the family Vibrionaceae (e.g., ASVs_ 4495, 4496). At T48, only four taxa were present in greater abundance in corals exposed to feces compared to control corals (Supplementary Table 19; log2 fold change: from 6.7 to 20.7) and included members from the genera *Propionigenium* (ASV_209), *Oceanospirillum* (ASV_5287) and the family Arcobacteraceae (ASVs_1360, 1368). At TF and under 26°C, only two taxa from the genera *Propionigenium* (ASV_210) and *Ruegeria* (ASV_2445) were present in greater abundance in corals exposed to feces compared to control corals (log2 fold change: from 20.5 to 20.7).

At T0 and under 30°C, only two ASVs from the family Vibrionaceae (ASV_4495) and the class Mollicutes (ASV_1646) were present in greater abundance in corals exposed to feces compared to controls (Figures 3B,C and Supplementary Table 20; log2 fold change: 21.9 to 24.4). However, at T24, this number reached 62 ASVs (Supplementary Table 20; log2 fold change: from 7.1 to 23.2) and included, among others, members from the family Clostridiaceae (e.g., ASVs_342, 355) and from the genera *Propionigenium* (ASVs_208-212), *Arcobacter* (e.g., ASVs_1327, 1330), *Photobacterium* (e.g., ASVs_4493, 4501) and *Vibrio* (ASVs_4577, 4579). At T48, the number of differentially abundant taxa in corals exposed to feces decreased to 8 ASVs compared to control corals (Supplementary Table 20; log2 fold change: from 19.9 to 21.1), and included members from the families Arcobacteraceae (ASVs_1316, 1318) and Vibrionaceae (ASVs_4532, 4620).

#### Effect of Temperature on Treatments Within Each Time Period

**FIGURE 4:**
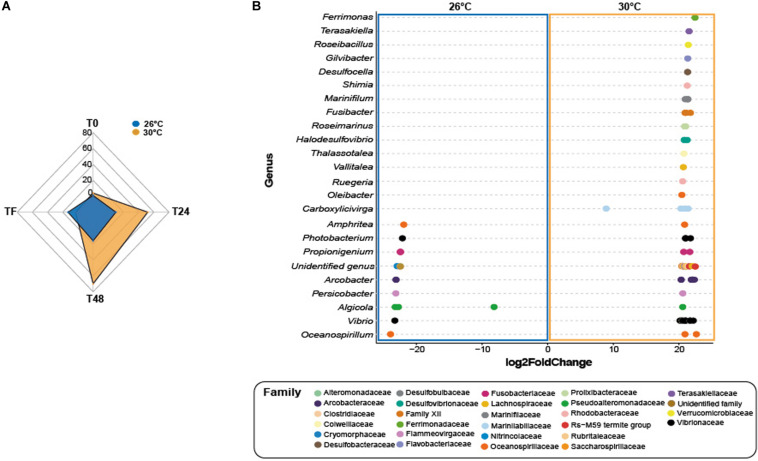
Differential abundance analysis based on DESeq2. **(A)** Number of differentially abundant ASVs observed at 26°C compared 30°C in corals exposed to fish feces exclusively. **(B)** The ASVs labeled as family and genus that differed significantly between 26°C and 30°C in the treatment Feces at time T48. Positive values are associated with differentially abundant taxa observed in corals exposed to the feces treatment at 30°C.

At T0, control corals showed greater abundance of eight taxa (Figure 4A and Supplementary Table 21; log2fold change: from −24.1 to −22.2), including members from the family Helicobacteraceae (ASV_1382), and the genera Candidatus Amoebophilus (ASVs_3008, 3009) and *Caedibacter* (ASV_5056-5058) at 26°C compared to 30°C. Only one ASV from the Mollicutes class was present in greater abundance at 30°C compared to 26°C in corals exposed to feces (Table S21; log2-fold change: 25.2), while four taxa from the genus *Vibrio* (e.g., ASVs_4594, 4599) were present in greater abundance at 26°C compared to 30°C (log2-fold change: from −24.4 to −22.7).

At T24, only one ASV from the genus *Ruegeria* (ASV_2441) was present in greater abundance in control corals at 30°C compared to 26°C (Table S22; log2-fold change: 21.5). Importantly, the number of differentially abundant taxa in corals exposed to feces increased to 62 ASVs at 30°C compared to 26°C (Figure 4A and Supplementary Table 22; log2-fold change: from 8.1 to 24.3). This included members from the families Lentisphaeraceae (ASV_150), Fusobacteriaceae (ASV_208), Clostridiaceae (e.g., ASVs_342, 363), Arcobacteraceae (e.g., ASVs_1330, 1333), Rhodobacteraceae (ASVs_1887, 2452, ASV_2446) and from the genera *Ferrimonas* (e.g., ASVs_4512, 4515), *Thalassotalea* (ASVS_5332, 5349), *Photobacterium* (e.g., ASVs_4497 4445) and *Vibrio* (e.g., ASVs_4563, 4577). Within the Vibrionaceae family, some sequences were closely affiliated to *Photobacterium rosenbergii*, *Vibrio ishigakensis*, *Vibrio harveyi*, *Vibrio alginolyticus* and *Vibrio vulnificus* (Supplementary Table 22). In contrast, ten taxa were present in greater abundance at 26°C compared to 30°C (Supplementary Table 22; log2-fold change: −23.5 to −8.1) and included taxa from the family Arcobacteraceae (e.g., ASVs_1363, 1369), and Vibrionaceae (ASV_4495) and from the genus *Ruegeria* (ASV_2442).

At T48 and within the feces treatment, 70 taxa were present in greater abundance at 30°C compared to 26°C (Figures 4A,B and Supplementary Table 22; log2-fold change: from 8.8 to 22.9), including ASVs from the families Lachnospiraceae (e.g., ASVs_323, 324), Clostridiaceae (e.g., ASVs_347, 348), Rhodobacteraceae (e.g., ASVs_1866, 1880), Desulfobacteraceae (ASV_2571), Desulfobulbaceae (ASV_2800), Fusobacteriaceae (ASVs_200), and genera *Halodesulfovibrio* (e.g., ASVs_2816, 2817), *Ferrimonas* (ASV_4512), *Vibrio* (e.g., ASVs_4574, 4581) and *Photobacterium* (ASVs_4498, 4532). Some sequences were closely affiliated to *Photobacterium rosenbergii*, *Vibrio parahaemolyticus*, *Vibrio harveyi*, *Vibrio hangzhouensis*, *Vibrio vulnificus* (Table S18). Conversely, 16 taxa showed greater abundance at 26°C compared to 30°C, such as Arcobacteraceae (ASVs_1330, 1360), Cyclobacteriaceae (ASV_3109), Vibrionaceae (ASVs_4621, 4623; ASV_4503), Pseudoalteromonadaceae (e.g., ASVs_4662, 4663).

At TF, corals exposed to feces showed greater abundance of only three taxa at 30°C compared to 26°C (Supplementary Table 22; log2fold change: from 22.4 to 26.2), including a member from the family Amoebophilaceae (ASV_3010). In contrast, 13 taxa were present in greater abundance at 26°C compared to 30°C (log2-fold change: from −23.4 to −21.9) and included, among others, ASVs from the families Fusobacteriaceae (ASVs_202, 204), Desulfobulbaceae (ASV_2806), Vibrionaceae (ASV_4495) and Ferrimonadaceae (ASV_4544).

### Exposure to Fish Feces Increases Coral Microbiome Community Variability

Temperature did not affect microbial community variability in control corals or corals exposed to feces throughout the experiment (Supplementary Tables 23, 24). However, there was a significant effect of the treatment (Feces vs Control) on community variability through time and within each temperature (Supplementary Figures 6, 7 and Supplementary Tables 25, 26). Under normal temperature (26°C) and at the beginning of the experiment (T0), corals exposed to feces showed similar values of bacterial community variability compared to control corals (Supplementary Figure 6; 0.16 ± 0.05; Supplementary Table 25; *p* = 0.44). Yet, corals exposed to feces showed greater values of community variability at T24, T48 and TF compared to control corals (Supplementary Table 25; *p* ≤ 0.01 for all tests). Under elevated temperature (30°C), corals exposed to feces exhibited greater values of community variability at T0, T48 and TF compared to control corals (Supplementary Figure 7 and Supplementary Table 26; *p* < 0.03 for all tests). In addition, time significantly affected bacterial community variability in control corals and corals exposed to feces during the exposure phase (Supplementary Figures 8, 9 and Supplementary Tables 27, 28). In control corals, community variability was greater at T24 compared to T0 (Supplementary Figure 8A and Supplementary Table 27; *p* = 0.02) and T48 compared to T24 (*p* = 0.01). Corals exposed to feces showed ∼2x greater values of community variability at T24 and T48 compared to T0 (Supplementary Figure 8B and Supplementary Table 28; *p* < 0.001 for all tests). During the recovery phase, while no change was observed in control corals (Supplementary Figure 9A and Supplementary Table 29), corals previously exposed to feces exhibited a decrease in community variability at TF compared to T48 (Figure 8B; Supplementary Table 30; *p* = 0.006).

### Indicator Species Associated With the Coral Microbiomes Closely Affiliated to Potential Coral Pathogens

BLASTn searches within the indicator species output (indicspecies results; Supplementary Table 31; Table 1) revealed the occurrence of nine specific ASVs that were closely related to potential coral pathogens. These taxa included *Photobacterium rosenbergii* (ASVs_4495, 4496, 4501; Table 1), *Vibrio harveyi* (ASV_4579, 4586, 4587), *Vibrio alginolyticus* (ASV_4589) and *Vibrio vulnificus* (ASV_4599, 4607). They were present in the microbiomes of corals exposed to feces within the first 48h of the experiment and under both temperatures (Table 1), but absent from control corals.

**TABLE 1:** Indicator species closely affiliated to potential pathogens across the coral dataset [Closely related taxa are associated with their percent identity (%), appropriate accession number and the experimental conditions (treatment, temperature, and time) in which they were identified].

**ASV Number(s)**	**Closely related species**	**Percent Identity (%)**	**Accession number**	**Conditions(s)**	**ASV found in Control**	**ASV found in *C. striatus* 16S fecal sample library (present study)**	**Taxon found in *C. striatu*s fecal metagenome sample (Ezzat et al., 2019)**
ASV_4495 ASVs_4496, 4501	*Photobacterium rosenbergii*	99.27 99.63	MN339950.1 MN339949.1	Feces-26°C-T0 Feces-30°C-T0 Feces-26°C-T0 Feces-26°C-T24h Feces-30°C-T24h	No	Yes No	No
ASV_4579 ASV_4586 ASV_4587	*Vibrio harveyi*	99.26 99.63 100	DQ995242.1 MT071645.1 MT071638.1	Feces-26°C-T0 Feces-26°C-T24h Feces-26°C-T48h Feces-30°C-T0 Feces-26°C-T0 Feces-26°C-T24h Feces-30°C-T24h Feces-26°C-T48h		No	Yes
ASV_4582 ASV_4623	*Vibrio ishigakengsis*	98.9 98.9	N339965.1 MN842811.1	Feces-26°C-T0 Feces-26°C-T24h Feces-30°C-T24h Feces-26°C-T0 Feces-26°C-T48h		Yes No	No
ASV_4589	*Vibrio alginolyticus*	99.63	MN210933.1	Feces-30°C-T24h		No	Yes
ASV_4599, 4607	*Vibrio vulnificus*	99.27	MT052658.1	Feces-26°C-T0 Feces-30°C-T24h		No	Yes

### Percentage of Tissue Recovery Affected by Elevated Temperature and Correlated With Relative Abundance of Specific Bacterial Taxa

**FIGURE 5:**
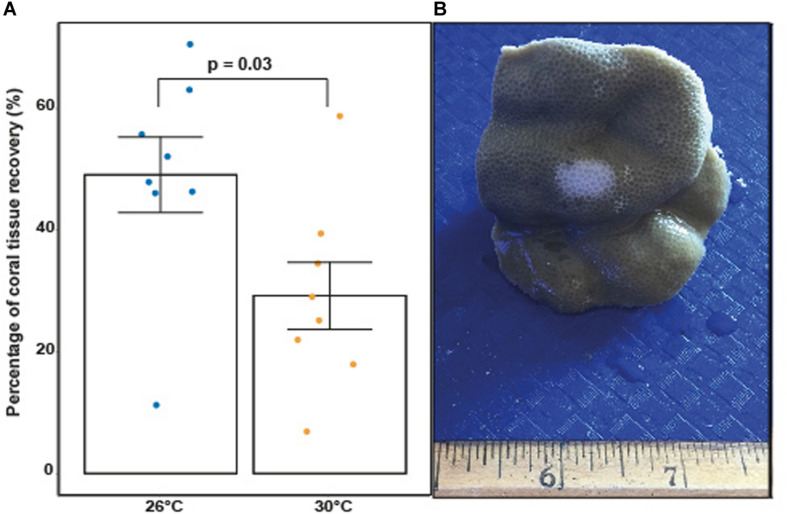
**(A)** Percentage of coral tissue recovery (%) in coral fragments previously exposed to fish feces at 26°C and 30°C at TF (1 week). *P*-value indicates statistical significance based on an ANOVA. **(B)** *Porites lobata* fragment with apparent sign of tissue loss and bleaching following removal of fecal pellet at T48 under elevated temperature (image: Leïla Ezzat).

All results presented in this section applied to corals exposed to fish feces only given that there were no areas of tissue bleaching observed on control corals. Elevated temperature significantly decreased the percentages of coral tissue recovery between T48 and TF in *P. lobata* exposed to fish feces (Figure 5 and Supplementary Table 32; *F*-value = 5.75; *p* = 0.031). Percentage of tissue recovery was 1.5 time lower at 30°C (29.22% ± 5.51%) compared to 26°C (49.23% ± 6.2%).

**FIGURE 6:**
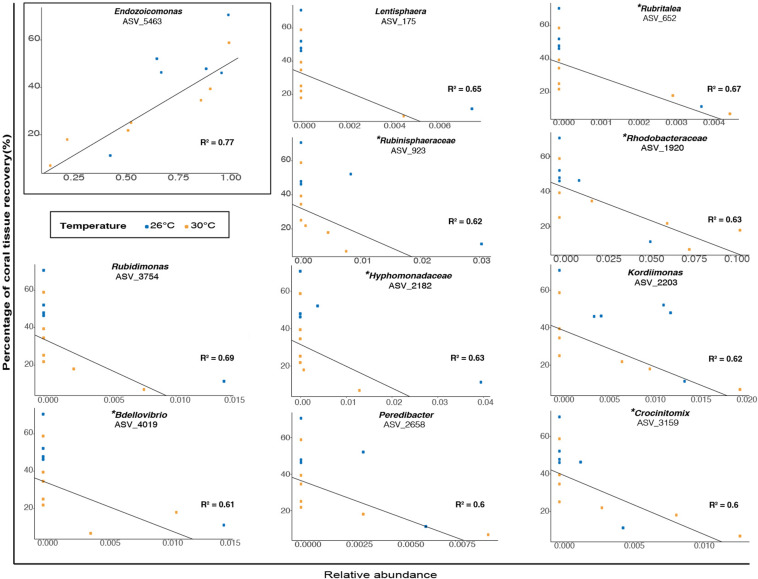
Relationship between the percentage of coral tissue recovery (%) and the relative abundance (proportion) of bacterial taxa (classified as genera or family) in microbiomes of corals previously exposed to fish feces at TF (1 week). Blue and orange dots represent ambient and elevated temperatures respectively. The presence of a specific bacterial taxon in surgeonfish feces is specified by the symbol (^∗^).

When examining association patterns between the relative abundance of individual ASVs and the percentage of coral tissue recovery, we found that the distribution of ranked coefficients of determination had a first discontinuity between its 1st and 2nd values, and a second clear discontinuity between its 11th and 12th values, i.e., for values of *R*^2^ > 0.59 (Supplementary Figure 10). We restricted investigation of biological relevance to 11 ASVs (Figure 6 and Supplementary Table 33), while acknowledging the possibility that associations may be specific to our particular dataset. Therefore, at the end of the experiment (TF), the percentage of tissue recovery was positively correlated with the relative abundance of one indicator taxon from the genus *Endozoicomonas* (Figure 6; ASV_5463; *R*^2^ = 0.77). In contrast, the percentage of tissue recovery was negatively related with the relative abundance of ten bacterial taxa from the genera *Rubidimonas* (Figure 6; ASVs_3754; *R*^2^ = 0.69), *Rubritalea* (ASV_ 652; *R*^2^ = 0.67), *Lentisphaera* (ASV_175; *R*^2^ = 0.54), *Kordiimonas* (ASV_2203; *R*^2^ = 0.62), Rubinisphaeraceae (ASV_923; *R*^2^ = 0.62), *Bdellovibrio* (ASV_4019; *R*^2^ = 0.61), *Crocinitomix* (ASV_3159; *R*^2^ = 0.6) and *Peredibacter* (ASV_2658; *R*^2^ = 0.6) as well as members from the families Hyphomonadaceae (ASV_2182; *R*^2^ = 0.63) and Rhodobacteraceae (ASV_1920; R^2^ = 0.63) – with some of these families and genera also present in fish fecal libraries (Ezzat et al., 2019 or present study) (Figure 6).

## Discussion

Coral-associated bacterial communities are sensitive to a variety of biotic and environmental stressors that can lead to microbial dysbiosis. Elevated temperature is among a number of environmental stressors that can favor shifts in bacterial communities and reduce immune function in reef-building corals. Moreover, one recently appreciated factor disrupting coral microbiomes is the deposition of fish feces on coral surfaces (Ezzat et al., 2019). Our findings suggest that the combination of surgeonfish feces and elevated temperatures created unique dynamics in *P. lobata* microbiomes compared to either stressor alone. Regardless of temperature, exposure of *P. lobata* to surgeonfish feces caused a significant shift in bacterial communities and resulted in increased alpha diversity and microbiome variability, all indicative of microbial dysbiosis. Although elevated water temperature did not lead to significant changes in alpha and beta diversity throughout the experiment, we noted a greater number of differentially abundant opportunists and potential pathogens in microbiomes of corals exposed to both feces and thermal stress. Among these taxa, some were associated with surgeonfish feces, suggesting that microbial enrichment and/or transmission from fish feces to corals occurs under, and may be facilitated by, elevated temperature. Furthermore, the impacts of fish feces and higher temperature on coral microbiomes appeared to inhibit wound healing in corals, as coral tissue percentages of coral tissue recovery at the site of feces deposition were significantly lower at 30°C compared to 26°C. Lesion recovery was negatively correlated with relative abundances of various bacterial taxa, including some found specifically in *C. striatus* feces. Our results suggest that increased sea surface temperature may exacerbate the effects of fish feces on coral microbiomes, ultimately altering *P. lobata* resilience.

### Fish Feces Increased Dysbiosis Susceptibility in *P. lobata* Regardless of Temperature

Deposition of fish fecal material on coral surfaces is a common biotic event on coral reefs that may result in significant source of nutrients for reef-building corals and other marine organisms (Robertson, 1982; Smriga et al., 2010). Yet, reef fishes harbor a diverse and abundant gut microbiota, including potential coral opportunists and bacterial pathogens (Garren et al., 2009; Smriga et al., 2010; Ezzat et al., 2019). Our findings demonstrate that, regardless of temperature, exposure of *P. lobata* to *C. striatus* feces resulted in an increase in ASV richness and diversity and a significant shift in bacterial community composition within the first 24h of the exposure phase compared to control corals. Patterns of elevated alpha diversity were previously associated with multiple abiotic and biotic stressors (i.e., increased sea surface temperature, ocean acidification, macroalgal competition, mechanical and biological wounding, McDevitt-Irwin et al., 2019), including interactions with fish feces (Ezzat et al., 2019). It should be noted that although statistically significant, the combined effect of fish feces exposure and time explains a relatively small amount of the total variance. That said, changes in bacterial community composition and elevated alpha diversity associated with *C. striatus* feces deposition coincided with increased relative abundance of members from the families Alteromonadaceae, Flammeovirgaceae, Rhodobacteraceae, Pseudoalteromonadaceae, Clostridiaceae, Desulfobulbaceae, Lentisphaeraceae and the genera *Photobacterium, Vibrio, Propionigenium, Thalassotalea and Arcobacter* in coral microbiomes. The presence of specific taxa in surgeonfish feces (i.e., families: Lentisphaeraceae, Lachnospiraceae, Oligoflexaceae, Desulfobulbaceae; genera: *Photobacterium*, *Vibrio*, *Propionigenium, Persicobacter*) and their absence in control corals within the exposure phase suggests potential mechanisms of bacterial enrichment and/or transmission from fish feces to coral surfaces. For instance, microbial lineages from the genera *Propionigenium*, *Photobacterium*, and *Vibrio*, as well as the families Lachnospiraceae and Desulfobulbaceae, are commonly found in the gut of marine fishes (Smriga et al., 2010; Givens, 2012; Miyake et al., 2015; Yamazaki et al., 2016; Smith et al., 2017). Interestingly, some of the aforementioned taxa, such as Vibrionaceae, Colwelliaceae, Clostridiaceae, Arcobacteraceae and Desulfobulbaceae, are often associated with stressed corals, diseased lesions or bleaching (Thompson et al., 2006; Weil et al., 2006; Sunagawa et al., 2009; Arotsker et al., 2015). Additionally, microbiomes of corals exposed to feces showed a significant decrease in the relative abundance of the potential beneficial symbiont Endozoicomonadaceae between T0 and T24 (from 71.65% to 27.3%) - an observation that aligns well with previous results on stressed corals (Morrow et al., 2015; Glasl et al., 2016; Ziegler et al., 2017; Pootakham et al., 2019; Maher et al., 2020).

These findings are in agreement with previous work exploring the effects of fish farm effluents on coral microbiota that documented similar shifts in bacterial communities, mainly explained by the strong prevalence of opportunistic lineages (i.e., *Desulfovibrio*, *Fusobacterium*) in microbiomes of corals exposed to elevated discharges of effluents (Garren et al., 2009). In addition, the present observations reinforce the conclusions of our recent work (Ezzat et al., 2019) and further confirm the potential for surgeonfish feces to act as vectors of microbial opportunists in corals. It is worth noting that corals exposed to *C. striatus* feces showed an overall increase in variability of bacterial communities during the exposure phase compared to control corals, regardless of temperature. These observations are consistent with previous studies demonstrating greater stochasticity and microbiome variability in a wide variety of organisms, including corals, when subjected to environmental and biotic stressors (i.e., Anna Karenina principle Zaneveld et al., 2017), and further support the case that *C. striatus* fecal pellets may enhance dysbiosis susceptibility within reef-building corals.

Despite one week exposure to temperature-induced thermal stress, *P. lobata* corals did not exhibit any significant changes in bacterial community composition and alpha diversity. These findings contrast with evidence suggesting that elevated sea surface temperature alone or its combination with other stressors (i.e., contact with macroalgae, nutrient enrichment) drive shifts in bacterial community composition or/and modulate alpha diversity in reef-building corals (Bourne et al., 2008; Mouchka et al., 2010; Zaneveld et al., 2016; Grottoli et al., 2018; Maher et al., 2019). However, other studies demonstrated stability of microbiomes alpha and/or beta diversity following exposure of corals to high temperatures (Salerno et al., 2011; Webster et al., 2016; Ziegler et al., 2017; McDevitt-Irwin et al., 2019; Rice et al., 2019b), including species from the genus *Porites*. These discrepancies among studies indicate a high variability in coral thermal tolerance threshold and microbiome flexibility following changes in environmental conditions. For instance, corals thriving in thermally variable environments that experience frequent episodes of thermal stress, such as the back reef areas in Mo’orea (Putnam and Edmunds, 2011), may exhibit a more stable microbiome throughout the stress events compared to heat-sensitive holobionts from cooler and more stable environments (Ziegler et al., 2017). That said, our results support the general belief that massive colonies from the genus *Porites* exhibit high physiological tolerance and effective immune response to heat stress (Loya et al., 2001; Adjeroud et al., 2009; Palmer et al., 2011; van Woesik et al., 2011; Barshis et al., 2018). More work is required to understand the role of microbial communities in shaping coral responses to thermal stress.

### Thermal Stress Exacerbated the Impact of Fish Feces on Coral Microbiomes

Elevated temperature alone did not lead to significant changes in bacterial community composition, but when paired with exposure to surgeonfish feces, we observed increased differential abundance of a variety of microbial taxa in corals – including potential opportunists and coral pathogens – compared to control corals at 30°C. For instance, 24 h exposure of corals to fish feces led to greater numbers of bacterial taxa, such as members from the genera *Propionigenium*, *Arcobacter*, *Persicobacter*, *Photobacterium* and *Vibrio, Ferrimonas* compared to controls under both temperatures. Importantly, these increases in taxa were considerably greater at 30°C compared to 26°C (i.e., 62 taxa at 30°C vs 25 taxa at 26°C, Figure 3). The absence of specific bacterial taxa in microbiomes of control corals (i.e., *Propionigenium*, *Persicobacter*, *Photobacterium*, and *Vibrio*), and their presence in moderate or relatively low abundance in corals exposed to feces and in surgeonfish feces samples suggests patterns of bacterial transmission from fish feces to corals. This was observed at both 26°C and 30°C, but exacerbated when temperatures were higher. Furthermore, we observed significant differences in the abundance of specific taxa when testing the effects of elevated temperature on coral microbiomes within each treatment (control and feces). While elevated temperature did not lead to increased abundance of bacterial taxa in control corals (26°C vs 30°C), we observed blooms of > 60 taxa in corals exposed to fish feces at 30°C compared to 26°C within the first 24 h of the exposure phase (Figure 4). This included taxa from the families Clostridiaceae, Arcobacteraceae, Rhodobacteraceae, Flammeorvirgaceae, Marinilabiliaceae, Vibrionaceae, Alteromonodaceae, Pseudoalteromonodaceae, Nitrincolaceae, Oceanospirillaceae, and Colwelliaceae. These observations showed that combination of both fish feces and increased sea surface temperatures resulted in unique microbial dynamics on coral surface when compared to either stressor alone.

Many of the bacterial taxa that bloomed on coral surfaces are known to respond to thermal and fecal stressors. For instance, members from the genera *Ruegeria, Arcobacter*, *Ferrimonas*, and the families Colwelliaceae and Clostridiaceae, often populate the microbiomes of corals and other marine organisms during thermal stress (Kimura et al., 1996; Simister et al., 2012; Gajigan et al., 2017). Evidence also suggests that the abundance of strains from the genera *Vibrio* and *Photobacterium* found in fish gut, corals and other fish food are exacerbated under ocean warming (Kelly, 1982; DePaola et al., 1994; Ben-Haim et al., 2003; Martinez-Urtaza et al., 2010; Vidal-Dupiol et al., 2011; Givens, 2012; Tout et al., 2015; Gajigan et al., 2017). Additionally, the gut of marine fishes includes micro-nutrients which may promote the growth of iron- and sulfate- or sulfur-reducing cells, including strains from the genera *Ferrimonas, Fusibacter* and members from the family Clostridiaceae in microbial communities of corals exposed to feces (Akagi and Campbell, 1962; Ravot et al., 1999; Nolan et al., 2010).

That said, the impact of these bacteria on coral microbiomes may vary, ranging from beneficial to potentially pathogenic. For example, two sequences from the genus *Ruegeria* (observed in microbial communities of corals exposed to feces under both temperatures) were closely related to *Ruegeria arenilitoris*, a bacterial species known to inhibit the growth of the coral pathogen *Vibrio coralliilyticus* (Miura et al., 2019). In contrast, taxa from the genera *Arcobacter*, *Persicobacter* and from the families Colwelliaceae and Clostridiaceae often populate the microbiomes of stressed and diseased corals as well as other marine organisms (Thompson et al., 2006; Kellogg et al., 2013; Sweet and Bythell, 2015; Ramees et al., 2017). Importantly, some sequences from the family Vibrionaceae exhibiting increased abundance at 30°C in corals exposed to feces compared to 26°C during the exposure phase were highlighted as indicator species of the feces treatment (Table 1) and closely affiliated to *Vibrio harveyi, Vibrio vulnificus, Vibrio ishigakensis, Vibrio alginolyticus and Photobacterium rosenbergii.* These taxa are often associated with particular diseases in humans, corals or other marine organisms such as fish as well as coral disease lesions and bleaching (DePaola et al., 1994; Thompson et al., 2005; Austin and Zhang, 2006; Luna et al., 2010; Meyer et al., 2019). Interestingly, the aforementioned five taxa, detected in different temperature regimes in our study, were simultaneously absent from control corals but present in either *C. striatus* feces 16S sample or metagenome (Ezzat et al., 2019), suggesting potential mechanisms of microbial transmission between surgeonfish feces and corals. It should be acknowledged that the aforementioned *Vibrio* sequences are equally good matches to several other *Vibrio* strains, confirming the limitations of sequencing the hypervariable V4 region of 16SrRNA gene for the precise identification of *Vibrio* species. Yet, the blooms of potential opportunists and pathogens in corals exposed to feces at elevated temperature are worrisome and raise concerns on potential subsequent impacts on coral health. As the frequency and duration of thermal stress related events may increase in the near future, common biotic interactions with reef fishes, such as release of fish fecal material, could turn harmful for corals. Whether these taxa actively affect *P. lobata* metabolism and resilience to stressors by acting as primary pathogens or simply exploit host resources when its immunity is compromised deserves further investigation.

Differences between microbiomes of control corals and corals exposed to feces were less pronounced by the end of the exposure phase (T48). We noted a drastic decrease in the differential abundance of potentially harmful taxa in microbiomes of corals exposed to feces compared to control at T48 in comparison to T24, regardless of temperature. This indicates a strong resilience of the coral holobiont, especially when both fish feces and elevated temperature interacted. These findings align well with previous studies demonstrating similar patterns in regulation of bacterial communities in corals exposed to stressors, including exposure to fish feces, fish farm effluents and thermal stress (Ritchie, 2006; Garren et al., 2009; Ezzat et al., 2019). The subtle changes observed could have resulted from an acclimative and transitory response of the coral host which lead to an altered state of its microbiome (van Oppen and Blackall, 2019). That said, even at T48, the number of differentially abundant ASVs when stressors were combined (high temperature and fish feces) still exceeded differences between control corals and corals that were exposed to either stressor alone. The persistence of a greater number of opportunistic and potential pathogens (i.e., *Vibrio*, *Photobacterium, Arcobacter*) in microbiomes of *P. lobata* exposed to feces at 30°C compared to 26°C suggests that temperature plays a critical role in exacerbating the effects of surgeonfish feces on coral microbiomes through time. Such taxa may have further contributed to detrimental health effects, such as apparent signs of bleaching and tissue mortality on surfaces of coral fragments previously exposed to fish feces – an observation already noted in the laboratory and in the field (Ezzat et al., 2019).

### Coral Tissue Recovery Correlated With the Relative Abundance of Specific Bacterial Taxa

Coral microbiomes largely recovered by the end of the experiment (TF, 1 week), as bacterial communities of corals exposed to feces shifted back toward their original composition and exhibited a significant reduction in community variability when compared to T48, regardless of temperature. The recovery of coral microbiomes was associated with a considerable increase in the relative abundance of Endozoicomonadaceae in microbiomes of corals exposed to feces at the end of the experiment (TF) compared to T48 (from 38.6% to 67.2%). Our findings parallel with a previous study demonstrating the resilience of Endozoicomonadaceae in corals following stressful conditions (Maher et al., 2020).

That said, signs of bleaching and tissue mortality were observed in the area of feces deposition at T48, regardless of temperature. Whether this was caused by opportunistic bacteria, hypoxic mechanisms due to the presence of feces, or combination of these factors deserves further investigation (Weber et al., 2012). Importantly, the percentage of coral tissue recovery between T48 and TF was three times lower at 30°C compared to 26°C. These observations align with previous studies demonstrating a slower rate of coral tissue regeneration following mechanical wounding or natural injury under elevated temperatures (Kramarsky-Winter and Loya, 2000; Lenihan and Edmunds, 2010; Bonesso et al., 2017; Rice et al., 2019b). Thermal stress may have impacted the metabolism and growth of *P. lobata*, thereby reducing the availability in nutritional resources for the holobiont – ultimately altering processes of cellular repair and tissue regeneration (Kramarsky-Winter and Loya, 2000; Bonesso et al., 2017).

Alternatively, one could hypothesize that the presence of potential opportunistic bacterial taxa could impede coral tissue recovery – with particularly negative impacts under elevated temperatures.

For instance, we observed negative relationships between the percentage of coral lesion recovery and the relative abundance of nine bacterial taxa at the end of the experiment and regardless of temperature. This included members from the families Saprospiraceae, Rhodobacteraceae, Bdellovibrionaceae, Kordiimonadaceae, Hyphomonadaceae, Lentisphaeraceae, Rubinisphaeraceae, Crocinitomicaceae, Bacteriovoraceae, Rubritaleaceae. Interestingly, six of these aforementioned bacterial families (i.e., Rhodobacteraceae, Bdellovibrionaceae, Hyphomonadaceae, Crocinitomicaceae, Rubritaleaceae, and Rubinisphaeraceae) were detected in relatively low abundance in *C. striatus* fecal 16S libraries (i.e., present study and Ezzat et al., 2019). Additionally, some of these bacterial lineages have been associated with stressed corals (i.e., Rhodobacteraceae; Pootakham et al., 2019), while other are known to populate coral exudates (i.e., Hyphomonadaceae, Bacteriovoraceae; Nelson et al., 2013; Welsh et al., 2015). Our results indicate that bacterial taxa commonly found in relatively low abundance in fish feces could potentially modulate lesion recovery in corals. It is however important to note that despite being statistically significant, the negative correlations are mostly driven by a small number of samples. Further studies are required to better understand these relationships and establish them with certainty. That said, we noted a strong positive relationship between the percentage of coral tissue recovery and the relative abundance of *Endozoicomonas*. Bacterial taxa from the genus *Endozoicomonas* - often associated with healthy coral colonies (Neave et al., 2016; Shiu and Tang, 2019) - are suggested to play important roles in the holobiont nutrient cycling and in the resilience of coral-associated bacterial communities (Bourne et al., 2016; Neave et al., 2016; Tandon et al., 2020). For instance, sequencing of the first endozoicomonal bacteria (i.e., *E. montiporae* CL-33^*T*^) from corals indicates potentials to prevent host mitochondrial dysfunction and favor gluconeogenesis during periods of stress (Ding et al., 2016). Moreover, evidence suggests that this particular taxon may provide protection to the photosymbionts against bleaching pathogens (Pantos et al., 2015). Although the present work demonstrates a clear relationship between the relative abundance of *Endozoicomonas* and the percentage of coral tissue recovery at the end of our experiment, our findings do not allow to establish whether *Endozoicomonas* has an active role in tissue recovery, or is rather a marker of coral tissue health. Further investigation is warranted to better understand the functional and ecological roles of *Endozoicomonas* and other specific bacterial symbionts in the microbial and physiological responses of reef-building corals to abiotic and biotic stressors.

## Conclusion

Our findings confirm the potential of surgeonfish to act as a vector of microbes within *P. lobata*, regardless of temperature. Although elevated water temperature alone did not lead to significant changes in microbial alpha and beta diversity, thermal stress exacerbated the impact of fish feces on the coral microbiome as we observed increased number of differentially abundant bacterial taxa in corals exposed to feces compared to controls at 30°C. Among these bacteria, an increasing number of potential pathogenic lineages were detected, including strains from the genera *Vibrio* and *Photobacterium* – previously found in surgeonfish feces. Although the microbiomes of corals exposed to feces began to look increasingly like those of control corals after 48h, the persistence of opportunistic taxa when both stressors interacted indicates that extreme temperatures may exacerbate the effects of fish feces on coral microbiomes. Interestingly, the percentages of lesion recovery related to fish feces deposition on corals were significantly lower at 30°C compared to 26°C at the end of the experiment. Importantly, these lower percentages of tissue recovery were negatively associated with greater relative abundance of specific taxa (i.e., Bdellovibrionaceae, Rhodobacteraceae, and Crocinitomicaceae), with some of them detected in relatively low abundance in surgeonfish feces. Our findings highlight the flexibility of *P. lobata* microbiomes in response to changing environmental and biotic conditions, and suggest the critical role of coral-associated bacterial communities in coral responses to human-induced stressors. However, as the frequency and duration of thermal stress related events may increase in the near future, the ability of coral microbiomes to recover from common biotic stressors such as deposition of fish feces may be greatly affected, compromising the health and resilience of their host. Our study provides evidence that common biotic interactions between reef fishes and corals, such as deposition of fish fecal material, could turn deadly for corals under anthropogenic stressors – ultimately impacting microbial dynamics in coral reefs. Future work assessing multiple scale response investigations (i.e., physiology, to microbes and genes) of coral species to different combinations of biotic and environmental disturbances will be important for understanding how human-induced stressors impact important biotic interactions in coral reefs.

## Data Availability Statement

Raw sequence data have been deposited in the Sequence Read Archive (SUB8734074) with NCBI BioProject accession no. PRJNA690899.

## Ethics Statement

The animal study was reviewed and approved by UC Animal Care and Use Committee (IACUC #196, 2016 – 2019).

## Author Contributions

LE, RVT, and DB designed the experiment. KM, KL, LE, and CC collected the samples. LE, KM, KL, SM, and CC performed the experiment. ES and CS performed laboratory work. LE analyzed the data. LE, CC, DB, and RVT wrote the paper. All authors contributed to the article and approved the submitted version.

## Conflict of Interest

The authors declare that the research was conducted in the absence of any commercial or financial relationships that could be construed as a potential conflict of interest.

## Footnotes

1 http://qiime2.org
