# Supplementary material for: Thermal Stress Interacts With Surgeonfish Feces to Increase Coral Susceptibility to Dysbiosis and Reduce Tissue Regeneration
Source: Front Microbiol. 2021 Mar 25;12:620458. doi: 10.3389/fmicb.2021.620458 (PMC8027513; doi:10.3389/fmicb.2021.620458)
Supplement: Supplementary file 2 [file Data_Sheet_1.docx]

***Supplementary material***

***Thermal stress interacts with surgeonfish feces to increase coral susceptibility to dysbiosis and reduce tissue regeneration***

Leïla Ezzat^1^, Sarah Merolla^2^, Cody S. Clements^3^, Katrina S. Munsterman^4^, Kaitlyn Landfield^1^, Colton Duran Stensrud^5^, Emily R. Schmeltzer^5^, Deron E. Burkepile^16^ and Rebecca Vega Thurber^5^

^1^Department of Ecology, Evolution and Marine Biology, University of California, Santa Barbara

^2^Coastal and Marine Science Institute, Bodega Marine Laboratory, University of California, Davis

^3^School of Biological Sciences, Georgia Institute of Technology

^4^Department of Ecology and Evolutionary Biology, University of Michigan

^5^Department of Microbiology, Oregon State University

^6^Marine Science Institute, University of California, Santa Barbara

**Text S1:** Results of differential abundance analyses - Comparison of the treatments within each time period and temperature

At T0, control corals showed a total of 10 taxa that increased in abundance compared to corals exposed to feces (Table S20; log2 fold change: from -25.7 to -22.2), including taxa from the families Endozoicomonadaceae (ASV_1382), Amoebophilaceae (i.e. ASVs_3008, 3009), Francisellaceae (ASVs_5056-5058) as well as Marinobacteraceae (ASV_5241). At T48, In contrast, control corals exhibited a greater abundance of six taxa compared to corals exposed to feces, such as four sequences from the family Endozoicomonaceae (genus Endozoicomonas: i.e. ASVs_5361, 5465). At 30°C and at T24, control corals showed increased abundance of six taxa such as members from the Rhodobacteraceae family (ASVs_2424, 2441) compared to corals exposed to feces (Table S21; log2 fold change: from -21.8 to -8.3).

**Figure S1.** Rarefaction curves of A) Species Richness and B) Shannon-Wiener indices according to the sampling depth. Red, blue and green dots are representative of the sample types (i.e. coral, water and fish fecal samples respectively)

**Figure S2.** Observed richness index during A) the exposure phase according to time (T0, T24, T48) and treatments (Control, Feces) and B) the recovery phase (T48, TF) according to treatment only. The horizontal line is the median, box height represents the interquartile ranges, whiskers extend 1.5x beyond the interquartile range, and dots are outliers. Different letters indicate significant differences in each panel based on ANOVA and pairwise comparisons (p < 0.05).

**Figure S3.** Shannon-Wiener index during A) the exposure phase according to time (T0, T24, T48) and treatments (Control, Feces) and B) the recovery phase (T48, TF) according to treatment only. The horizontal line is the median, box height represents the interquartile ranges, whiskers extend 1.5x beyond the interquartile range, and dots are outliers. Different letters indicate significant differences in each panel based on ANOVA and pairwise comparisons (p < 0.05).

**Figure S4.** Principal coordinates analysis of Bray-Curtis dissimilarity matrix of *P. lobata* corals exposed to the different treatments (Control, Feces) during the exposure phase (T0, T24, T48)


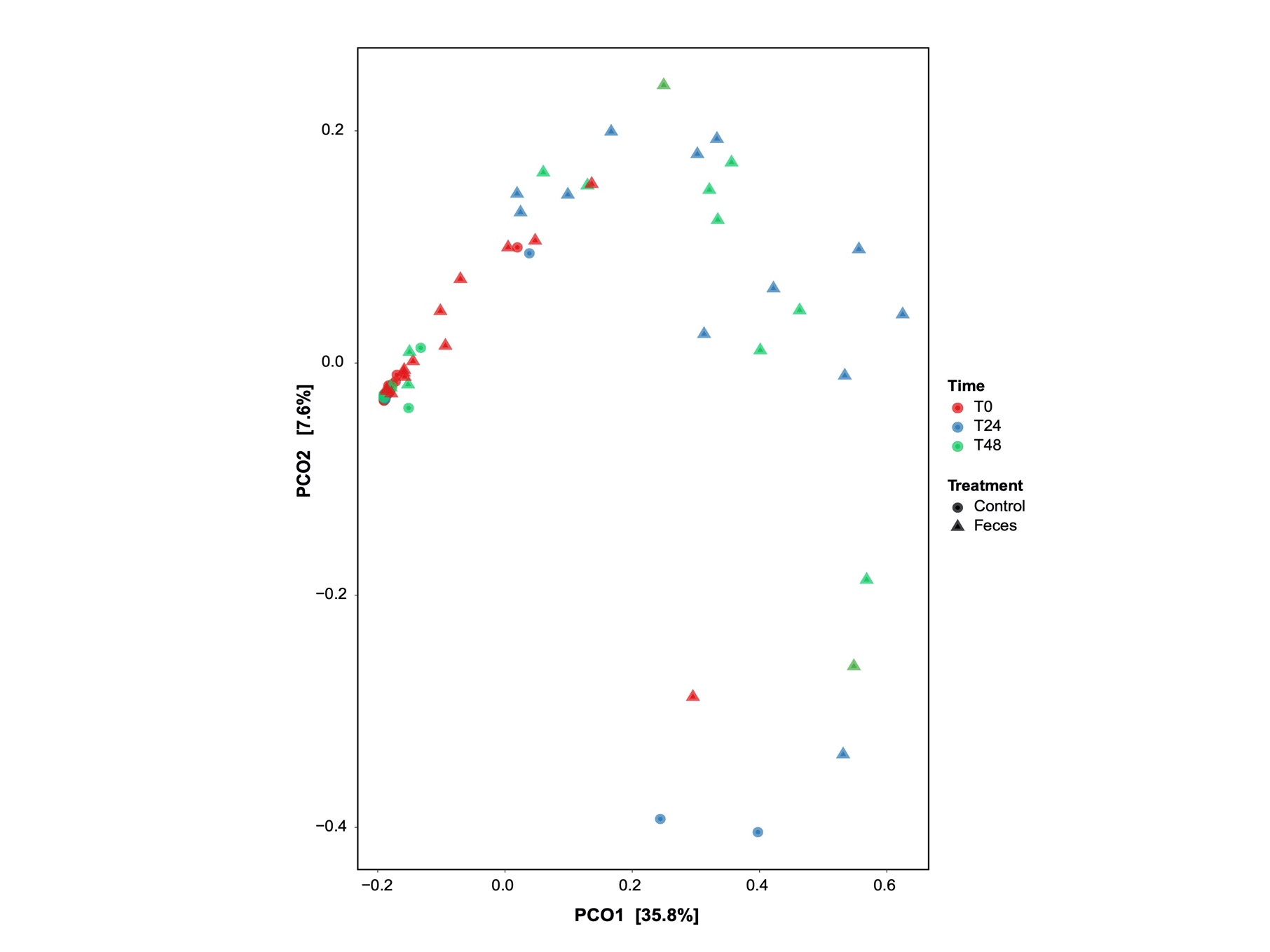


**Figure S5.** Principal coordinates analysis of Bray-Curtis dissimilarity matrix of *P. lobata* corals exposed to the different treatments (Control, Feces) during the recovery phase (T48, TF)

**
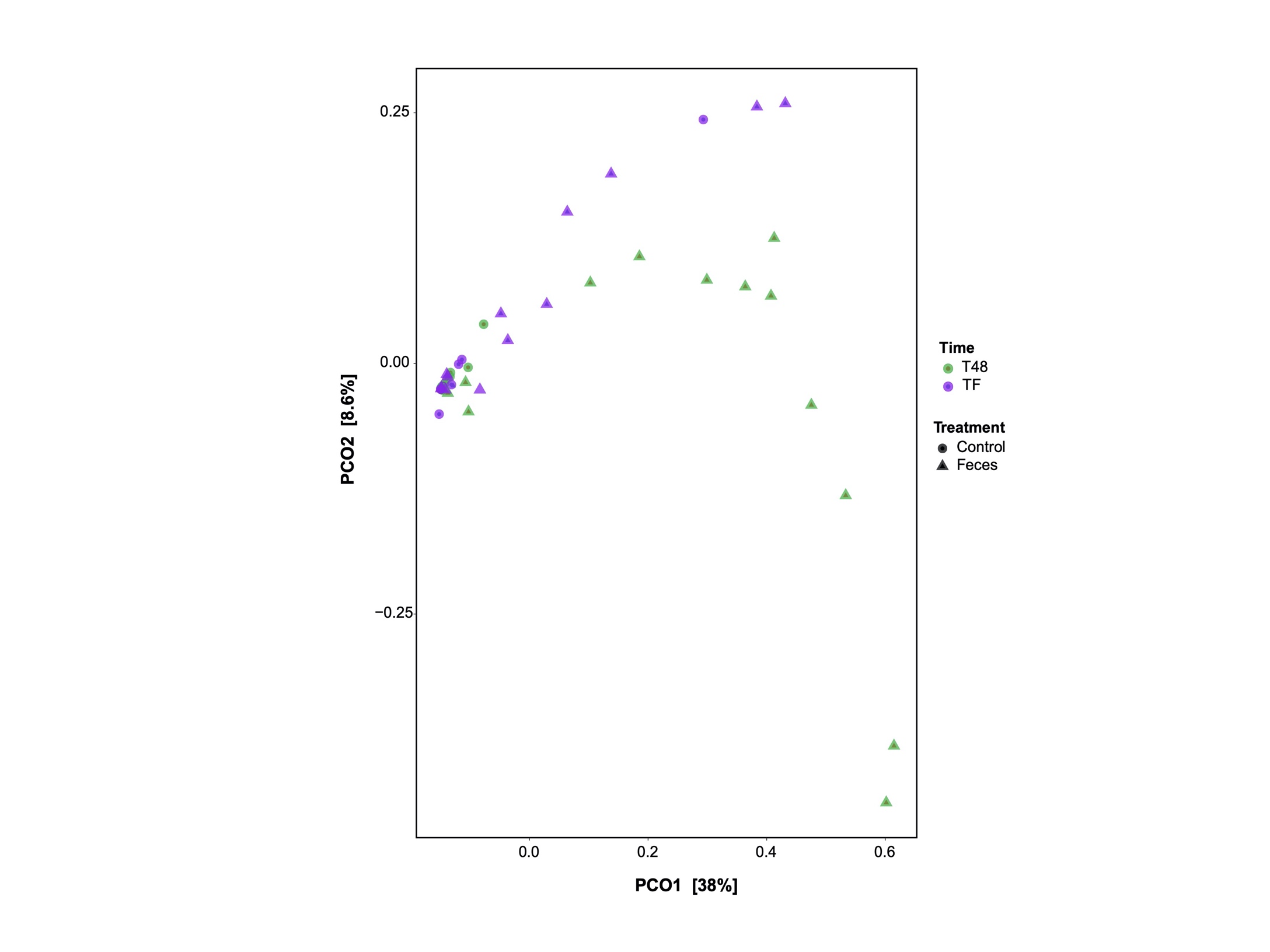
**

**Figure S6.** Effect of treatment (Feces vs Control) on bacterial community variability at 26°C within each time period (T0, T24, T48, TF)

**Figure S7.** Effect of treatment (Feces vs Control) on bacterial community variability at 30°C within each time period (T0, T24, T48, TF)

**Figure S8.** Effect of time on bacterial community variability during the exposure phase within treatment A) control and B) feces

**Figure S9.** Effect of time on bacterial community variability during the recovery time within treatment A) control and B) feces

**Figure S10.** Association patterns between the relative abundance of individual ASVs and the percentage of coral tissue recovery. Distribution of ranked coefficients of determination (R^2^) with two discontinuities between the 1st and 2nd values, and between its 11th and 12th values.

**
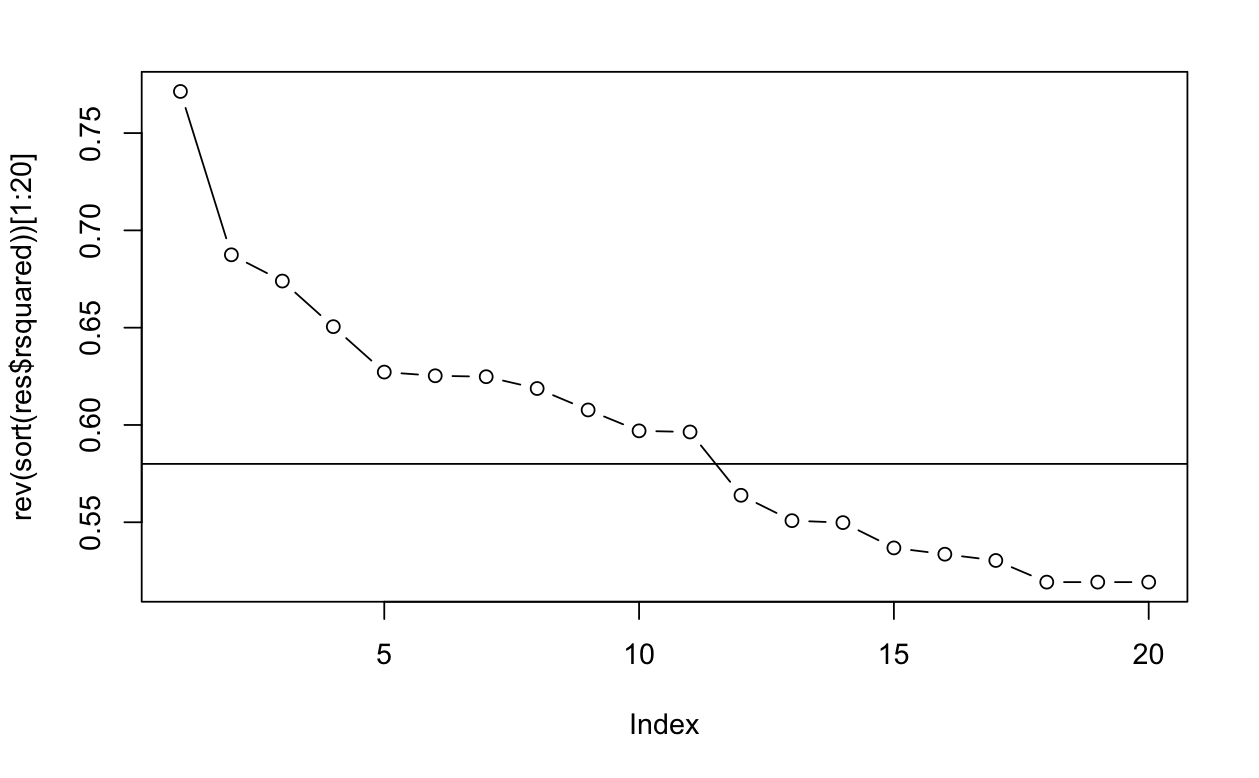
**
